# A Cross-Sectional and Longitudinal Study of Pain among Middle-Aged and Older Adults in Thailand

**DOI:** 10.1155/2023/1158899

**Published:** 2023-03-09

**Authors:** Supa Pengpid, Karl Peltzer

**Affiliations:** ^1^Department of Health Education and Behavioral Sciences, Faculty of Public Health, Mahidol University, Bangkok, Thailand; ^2^Department of Public Health, Sefako Makgatho Health Sciences University, Pretoria, South Africa; ^3^Department of Healthcare Administration, College of Medical and Health Science, Asia University, Taichung, Taiwan; ^4^Department of Psychology, University of the Free State, Bloemfontein, South Africa; ^5^Department of Psychology, College of Medical and Health Science, Asia University, Taichung, Taiwan

## Abstract

**Objective:**

The present study aimed to assess the prevalence and risk factors of pain among ageing adults in Thailand.

**Methods:**

Cross-sectional and longitudinal data were analysed from two consecutive national waves of the Health, Aging, and Retirement in Thailand (HART) study in 2015 and 2017. The dependent variable pain was defined as moderate or severe pain in any of the 13 areas of the body over the past month. Independent variables included sociodemographic factors, health risk behaviour, physical and mental health conditions, and healthcare utilization.

**Results:**

The baseline or cross-sectional sample consisted of 5,616 participants (≥45 years), and the follow-up or incident sample consisted of 2,305 participants. The proportion of pain in the cross-sectional/baseline sample was 36.0%, and in the incident/follow-up sample 39.9%. In the cross-sectional/baseline multivariable model, poor self-reported mental health, sleep problem, arthritis or rheumatism, brain disease and/or psychiatric problems, lung disease, use of hospital in-patient, conventional out-patient, and traditional medicine practitioners were positively associated with pain. In the incident/follow-up multivariable model, older age, Buddhist religion, class I obesity, poor self-reported mental health, hospital in-patient, private clinic out-patient, and use of a practitioner of traditional medicine were positively associated with pain. Male sex and higher education were negatively associated with both cross-sectional and incident pain.

**Conclusions:**

More than one-third of older adults in Thailand had past month moderate or severe pain. Risk factors of pain from cross-sectional and/or incident analysis included older age, female sex, lower education, obesity, poor self-reported mental health, sleep problem, arthritis or rheumatism, brain disease and/or psychiatric problems, lung disease, and conventional and traditional healthcare utilization.

## 1. Introduction

The population in Thailand is rapidly ageing, increasing the health burden of older adults [1]. In the general population, pain is a common symptom, comorbid with clinical conditions, and the prevalence ranges from 10% to 60% [2]. Pain has been redefined by the International Association for the Study of Pain as “An unpleasant sensory and emotional experience associated with, or resembling that associated with, actual or potential tissue damage [3].”

Data from adults in the US even have shown an increase in noncancer pain from 32.9% in 1997/1998 to 41.0% in 2013/2014 [4]. In middle-income countries, past 12-month mild, moderate, or severe pain was reported by 14.3% in Brazil, 6.2% in China, and 18.3% in Russia [5]. In the 2008 National Health and Wellness Survey in the UK, France, Italy, Germany, and Spain, the prevalence of moderate or severe pain in the adult population in the past month was 16.6% [6]. In a multicountry study among older adults, 39.5% developed pain between baseline and 5 years' follow-up [2]. In a national study that included people aged 60 and older in China, 32.5% reported having pain [7]. We lack incident and cross-sectional data on pain in general populations, such as in Southeast Asia and Thailand [2], which led to this study.

As reviewed by Raggi et al. [2], risk factors for pain may include sociodemographic factors such as lower education, female sex, older age, and mental health problems, including sleep problems, depressive symptoms, chronic conditions, such as stroke, diabetes, and obesity, and health risk behaviours, such as physical inactivity and smoking. In the multicountry study, cross-sectional risk factors for pain included obesity, female sex, and vigorous physical activity, and incident risk factors included walking difficulties, poor self-rated health, and sleep problems [2].

In addition, pain has been found to increase healthcare utilization. In a study among older adults in China, the pain was associated with an increased use of herbal medicine and over-the-counter drugs [7], in a study among adults in France, the pain was associated with more visits from healthcare providers, including emergency room visits and hospitalizations [8], and among adults with pain in a multicountry study (Brazil, China, Russia, Japan, USA, and developed European Union), physician visits, hospitalizations, and emergency room visits increased [5]. Considering the impact of pain on the life of older people, it is important to understand the pattern of pain characteristics and its risk factors, as well as healthcare utilization in Southeast Asia, including Thailand. The results may have implications for improving healthcare for patients with pain. Therefore, the objective of this cross-sectional and longitudinal study was to assess the prevalence and risk factors of pain in a national population-based sample of ageing adults in Thailand.

## 2. Methods

### 2.1. Study Design, Setting, and Participants

Cross-sectional and longitudinal national data were analysed from two consecutive surveys of the Health, Aging, and Retirement in Thailand (HART) study in 2015 and 2017 [9]. In a national sample, one household member (≥45 years) (inclusion criteria) was randomly selected by applying a multistage sampling process [9]; detailed procedures have been published [10]. The sample size of the 2015 survey was 5,616, and the sample of the 2017 survey was 3,708 participants, 1,908 were lost to follow-up, and the response and retention rate was 72.3% and 66.03%, respectively [11]. At baseline, a paper and pencil (PAPI) questionnaire were used, and at follow-up computer-assisted personal interviewing (CAPI) [9]. The Ethics Committee on Human Research of the National Institute of Development Administration, ECNIDA (ECNIDA 2020/00012), approved the study, and participants gave written informed consent [11]. We use Epi Info Version 7.2.2.6 for the calculation of the population survey sample size, prevalence of the previous study 32.5% pain [7], acceptable margin of error = 5, designing effect 1, at confidence level 99.99%, and the minimum sample required is 1327.

### 2.2. Measures

Outcome Variable Pain

HART examines past-month pain in 13 body parts (“the head, shoulders, arms, wrists, fingers, chest, abdomen, back, hips, legs, knees, ankles, and toes”) by asking the following question, “Did you feel any pain or ache in the following body parts in the last month?” Response options were none, mild, moderate, or severe. We defined moderate or severe pain in the past 4 weeks in one or more of the 13 body parts as “pain.” Cronbach alpha for the pain measure was 0.81 and 0.82 in wave 1 and wave 2, respectively.

*Healthcare utilisation* was assessed with utilization of the following public and private healthcare types: (1) public healthcare: hospital admission, hospital out-patient in the past two years (district hospitals provide primary healthcare (PHC), and secondary care and regional/general hospitals provide tertiary and other specialized care), a health center in the past two years (provides PHC services), medical home visit in the past two years, and medical check-up in the past year, (2) private healthcare: private clinic in the past two years (mostly curative services), and (3) traditional medicine doctor in the past two years (is mostly private but can also be public health service) (Yes/No) [10, 12].

*Sociodemographic variables* included income quartile, education, sex, age, and religion [13].

*Tobacco smoking*, “Have you ever smoked cigarettes?” (responses were “1 = yes, and still smoke now, 2 = yes, but quit smoking, and 3 = never”).

*Alcohol use*, “Have you ever drunk alcoholic beverages such as liquor, beer, or wine?” (response options: “1 = yes, and still drinking now, 2 = yes, but do not drink now, and 3 = never”).

*Physical activity* was defined as “none = inactivity, 1–149 min/week = low activity, and ≥150 min/week = high activity” [14].

*Body mass index* (BMI) was classified into “underweight (<18.5 kg/m^2^), normal weight (18.5–22.9 kg/m^2^), overweight (23–24.9 kg/m^2^), class I obesity (25–29.9 kg/m^2^), and class II obesity (30 kg/m^2^)” based on self-reported weight and height [15].

*Probable depression* (≥10 scores) was measured using the Center for Epidemiologic Studies Depression (CES-D-10) scale [16]; Cronbach alpha 0.78.

Self-reported mental health status: “In general, how would you rate your mental health status?” Responses were rated from “0 = very poor to 100 excellent,” and poor mental health was defined as “0 to less than 80 and good mental health as 80–100” [11].

*Sleep problem* was defined as “almost always or often (versus sometimes or very rarely or never) having trouble falling asleep/insomnia in the past week” [9].

*Chronic diseases* (diagnosed by a healthcare provider) included diabetes, hypertension, arthritis or rheumatism, emphysema, emotional/nervous or psychiatric illness, lung diseases, Alzheimer's disease, cardiovascular diseases, brain diseases, kidney diseases, heart disease, and heart failure [9].

*Functional disability* was classified as being unable to do any of the four activities of daily living (ADL) (eating, bathing, dressing, and washing) [17] (Cronbach's *α* 0.94).

### 2.3. Statistical Analysis

Descriptive statistics were used to describe cross-sectional and incident pain by demographic and health status factors. To test for differences in proportions, Pearson chi-square tests were applied. The first Poisson regression model estimated prevalence ratios (PRs) and confidence intervals (CIs) for cross-sectional pain prevalence, and the second model compared the baseline sample without pain with incident pain (developed pain at follow-up). Variables significant at *p* < 0.1 in bivariate analyses were subsequently incorporated into the multivariable models. *p* ≤ 0.05 was accepted as statistically significant. Statistical analyses were carried out using StataSE 15.0 (College Station, TX, USA); only complete cases were included in the analyses.

## 3. Results

### 3.1. Participants

The baseline or cross-sectional sample consisted of 5,616 individuals (45 years and older, 66 years median age), and the follow-up or incident sample consisted of 2,305 participants. The prevalence of pain in the cross-sectional/baseline sample was 36.0%, and the prevalence of incident pain (those who had pain at follow-up without pain at baseline) was 39.9%. In the baseline sample, most frequently moderate or severe pain was reported in the legs (19.0%), knees (18.6%), and back (11.8%), followed by hips (8.6%), arms (7.2%), shoulders (6.5%), head (5.5%), ankles (4.0%), wrists (3.1%), abdomen (3.0%), toes (2.1%), chest (1.9%), and fingers (1.8%). In the cross-sectional sample, binary analysis showed that sex, age, education, income, probable depression, body mass index, alcohol use status, self-reported mental health status, arthritis or rheumatism, sleep problem, kidney disease, hypertension, cardiovascular disease, diabetes, lung disease, functional disability, and brain disease or psychiatric problems differed significantly between persons with pain and without pain. In the incident population, binary analysis showed that sex, age, education, religion, income, smoking, physical activity, self-reported mental health status, hypertension, and diabetes differed significantly between persons with and without pain (see Table 1).

Table 2 shows the utilization of healthcare by cross-sectional and incident pain. Binary analysis in the cross-sectional population showed that all four types of healthcare utilization differed significantly between people with pain and without pain, and the binary analysis in the incident sample found that hospitalization, hospital out-patient, private clinic out-patient, and use of traditional medicine practitioner differed significantly between people with overall pain and without overall pain, as well as a leg, back, and knee pain (see Table 2).

### 3.2. Associations with Pain in Cross-Sectional Analysis

In the multivariable model, poor self-reported mental health (aPR: 1.49, 95% CI: 1.29 to 1.72), sleep problem (aPR: 1.99, 95% CI: 1.67 to 2.37), arthritis or rheumatism (aPR: 3.29, 95% CI: 2.36 to 4.60), lung disease (aPR: 1.94, 95% CI: 1.03 to 3.63), brain disease and/or psychiatric problems (aPR: 2.11, 95% CI: 1.12 to 3.96), hospital in-patient (aPR: 1.88, 95% CI: 1.55 to 2.28), hospital out-patient (aPR: 1.27, 95% CI: 1.10 to 1.47), private clinic out-patient (aPR: 1.51, 95% CI: 1.20 to 1.90), and use of traditional medicine practitioner (aPR: 2.41, 95% CI: 1.60 to 3.63) were positively associated, and male sex (aPR: 0.77, 95% CI: 0.67 to 0.88) and higher education (aPR: 0.63, 95% CI: 0.53 to 0.76) were inversely associated with cross-sectional pain (see Table 3).

### 3.3. Associations with Incident Pain

In the final multivariable model, older age (aPR: 1.02, 95% CI: 1.01 to 1.03), Buddhist religion (aPR: 1.85, 95% CI: 1.26 to 2.73), obesity class I (aPR: 1.28, 95% CI: 1.01 to 1.62), poor self-reported mental health (aPR: 1.27, 95% CI: 1.02 to 1.58), hospital in-patient (aPR: 1.75, 95% CI: 1.31 to 2.35), private clinic out-patient (aPR: 1.59, 95% CI: 1.22 to 2.07), and use of traditional medicine practitioner (aPR: 2.57, 95% CI: 1.61 to 4.09) were positively associated, and male sex (aPR: 0.80, 95% CI: 0.66 to 0.99) and higher education (aPR: 0.65, 95% CI: 0.51 to 0.83) were inversely associated with incident pain (see Table 4).

## 4. Discussion

It appears that this is the first study that assessed the prevalence and risk factors of pain among ageing adults (≥45 years) in a national household survey in Thailand in 2015 and 2017. The prevalence of moderate or severe pain in the cross-sectional population was 36.0% and in the incident population 39.9%. This result is similar to the prevalence of pain among older adults in China (32.5%) [7], among adults in the USA (32.9%–41.0%) [4], and in a multicountry study among older adults (39.5% incident pain) [2], but higher than among adults in Brazil, China, and Russia (ranging from 6.2% to 18.3% past 12-month mild, moderate, or severe pain, versus 70% mild, moderate, or severe past month pain in our study, analysis not shown) [5], and among adults in the UK, France, Italy, Germany, and Spain (16.6% moderate or severe past month pain) [6]. Some of these differences may be explained by social and cultural differences, as well as differences in the measurement or definition of pain.

Risk factors of pain from cross-sectional and/or incident analysis included older age, female sex, lower education, Buddhist religion, obesity class I, poor self-reported mental health, sleep problem, arthritis or rheumatism, brain disease and/or psychiatric problems, lung disease, hospital in-patient, conventional out-patient, and traditional medicine practitioner use. Our results are in line with former research in terms of older age [18], females [2, 5, 7, 18], and lower education [19, 20]. It is possible that older adults develop more physical conditions, which in turn may cause more pain in the body [18]. Compared to women, men may underreport pain due to internalized masculinity norms [18, 21]. Older adults with lower education and, in univariable analysis, lower income had a higher prevalence of pain. General education and access to financial resources can improve general health and reduce pain through health-behavioural, medical, and social factors [20]. Compared to Muslims or others, Buddhists had a higher prevalence of pain. Similarly, in a study in Thailand [22], the proportion of knee pain was lower in Muslims than in Buddhists, which may relate to differences in religious practices (“Muslims pray since childhood by forcing the knees into deep flexion, stretching the soft tissue surrounding the knee, and decrease stiffness and contact pressure of the articular cartilage”).

Consistent with previous research [2], we found that modifiable risk factors, such as obesity, poor self-reported mental health status, and sleep problems, increased the odds of pain. Obesity can contribute to pain through mechanical and inflammatory processes [2, 23]. Weight loss and sleep behaviour modification and mental health promotion can be used to address these modifiable risk factors [2]. In addition, physical exercise was found to be protective against pain in the univariable model. Although some studies show a bidirectional association between physical activity and knee pain [24]. On the other hand, some other studies found a bidirectional association between pain and sleep problems [25] and poor mental health (depressive symptoms) [26].

Furthermore, in agreement with previous research [2, 5], having certain comorbidities, such as arthritis [7] and lung diseases, increased the odds of pain. Older adults with arthritis are prone to pain disability [27], and pain is a common problem among patients with interstitial lung disease [28]. In addition, in univariable analyses, other noncommunicable diseases (NCDs), such as hypertension and diabetes were associated with pain. Given the large burden of NCDs in Thailand and the region, it may be important to synergistically target NCDs and pain relief interventions [7]. Moreover, in line with previous findings [5, 7, 8], pain increased hospital admission, conventional, and traditional medicine out-patient care visits. More research is needed to explore the specific pain management strategies, for example for the most frequently occurring pains related to the legs, knees, and back, by the different types of healthcare providers in Thailand. For example, a “Thai Medicinal Plant-4 (TMP-4) cream made up of *Garcinia mangostana* peel, *Sesamum indicum* seeds, *Glycine max* (L.) Merr. Seeds, and *Centella asiatica* leaves were not inferior to diclofenac gel in relieving osteoarthritic knee pain.” [29].

Study limitations include the evaluation of the self-report of all study variables. Furthermore, chronic pain and care or treatment modalities for pain were not assessed. Furthermore, the survey excluded institutionalised persons.

In conclusion, more than one-third of older adults in Thailand had past month moderate or severe pain. Risk factors of pain from cross-sectional and/or incident analysis included older age, female sex, lower education, Buddhist religion, obesity class I, poor self-reported mental health, sleep problem, arthritis or rheumatism, brain disease and/or psychiatric problems, lung disease, hospital in-patient, conventional out-patient, and traditional medicine practitioner use. The findings of this study may guide healthcare professionals and clinicians in improving pain management strategies. Additional investigations are indicated to assess chronic pain and types of pain management strategies by older adults in Thailand.

## Figures and Tables

**Table 1:** Demographic factors and health status by cross-sectional and incident pain.

Variables	Subcategory	Cross-sectional pain		*p* value	Incident pain		*p* value
		No	Yes		No	Yes	
All		3593 (64.0)	2023 (36.0)		1385 (60.1)	919 (39.9)	

Age (in years)	45–54	779 (70.5)	326 (29.5)	<0.001	303 (66.3)	154 (33.7)	<0.001
	55–64	974 (64.9)	526 (35.1)		403 (63.0)	237 (37.0)	
	65–74	858 (62.6)	512 (37.4)		346 (59.1)	239 (40.9)	
	75 or more	982 (59.8)	659 (40.2)		334 (53.6)	289 (46.4)	

Sex	Female	1772 (60.5)	1158 (39.5)	<0.001	647 (56.2)	504 (43.8)	<0.001
	Male	1821 (67.8)	865 (32.2)		739 (64.0)	415 (36.0)	

Education	None	201 (55.4)	162 (44.6)	<0.001	62 (51.2)	59 (48.8)	<0.001
	Elementary	2607 (61.8)	1610 (38.2)		1008 (58.1)	726 (41.9)	
	>Elementary	769 (75.7)	247 (24.3)		312 (70.3)	132 (29.7)	

Marital status	Not married	1489 (63.3)	863 (36.7)	0.403	522 (57.9)	380 (42.1)	0.080
	Married/cohabiting	2098 (64.4)	1160 (35.6)		862 (61.5)	539 (38.5)	

Religion	Muslim or other	244 (60.8)	157 (39.2)	0.182	125 (69.4)	55 (30.6)	0.008
	Buddhist	3342 (64.2)	1866 (35.8)		1259 (59.3)	864 (40.7)	

Income quartile	Low	830 (59.2)	573 (40.8)	<0.001	318 (62.8)	188 (37.2)	0.012
	Lower middle	848 (61.5)	531 (38.5)		313 (55.7)	249 (44.3)	
	Upper middle	902 (64.6)	517 (36.4)		358 (58.1)	258 (41.9)	
	High	1013 (71.6)	402 (28.4)		397 (63.9)	224 (36.1)	

Alcohol use	Never	2893 (63.9)	1637 (36.1)	0.028	1087 (58.9)	758 (41.1)	0.056
	Past	232 (59.3)	159 (40.7)		102 (65.8)	53 (34.2)	
	Current	468 (67.3)	227 (32.7)		197 (64.6)	108 (35.4)	

Smoking tobacco	Never	2875 (64.1)	1608 (35.9)	0.085	1075 (58.4)	766 (41.6)	<0.001
	Past	253 (59.4)	173 (40.6)		102 (62.6)	61 (37.4)	
	Current	465 (65.8)	242 (34.2)		209 (69.4)	92 (30.6)	

Physical activity	None	2166 (64.2)	1210 (35.8)	0.320	802 (58.9)	560 (41.1)	0.030
	1–149 min./week	852 (62.5)	511 (37.5)		328 (59.0)	228 (41.0)	
	≥150 min./week	575 (65.6)	302 (34.4)		256 (66.1)	131 (33.9)	

Body mass index	Normal	1270 (66.4)	642 (33.6)	0.010	490 (60.9)	314 (39.1)	0.281
	Underweight	347 (62.1)	212 (37.9)		140 (63.1)	82 (36.9)	
	Overweight	663 (65.8)	344 (34.2)		256 (60.4)	168 (39.6)	
	Obesity class I	773 (63.0)	454 (37.0)		277 (56.1)	217 (43.9)	
	Obesity class II	197 (51.6)	154 (43.9)		71 (57.7)	52 (42.3)	

Probable depression	No	2979 (65.8)	1549 (34.2)	<0.001	1162 (60.5)	760 (39.5)	0.772
	Yes	336 (52.5)	304 (47.5)		123 (59.4)	84 (40.6)	

Sleep problem	No	3141 (67.8)	1493 (32.2)	<0.001	1226 (60.8)	792 (39.2)	0.075
	Yes	409 (44.7)	507 (55.3)		143 (55.0)	117 (45.0)	

Self-reported mental health	Good	2596 (67.6)	1244 (32.4)	<0.001	1040 (61.3)	656 (38.7)	0.021
	Poor	924 (56.1)	723 (43.9)		316 (55.8)	250 (44.2)	

Arthritis or rheumatism	No	3520 (65.3)	1872 (34.7)	<0.001	1358 (60.2)	897 (39.8)	0.546
	Yes	73 (32.6)	151 (67.4)		28 (56.0)	22 (44.0)	

Hypertension	No	2435 (66.4)	1230 (33.6)	<0.001	944 (61.6)	588 (38.4)	0.040
	Yes	1158 (59.4)	793 (40.6)		442 (57.2)	331 (42.8)	

Diabetes	No	3080 (64.6)	1687 (35.4)	0.019	1207 (61.3)	762 (38.7)	0.005
	Yes	513 (60.4)	336 (39.6)		179 (53.3)	157 (46.7)	

Cardiovascular disease	No	3443 (64.5)	1896 (35.5)	<0.001	1331 (60.4)	872 (39.6)	0.190
	Yes	150 (54.2)	127 (45.8)		55 (53.9)	47 (46.1)	

Kidney diseases	No	3536 (64.2)	1975 (35.8)	0.037	1369 (60.4)	899 (39.6)	0.076
	Yes	57 (54.3)	48 (45.7)		17 (45.9)	20 (54.1)	

Brain diseases and psychiatric problems	No	3568 (64.3)	1983 (35.7)	<0.001	1378 (60.2)	912 (39.8)	0.590
	Yes	25 (38.5)	40 (61.5)		8 (53.3)	7 (46.7)	

Lung diseases	No	3570 (64.1)	1997 (35.9)	0.013	1376 (60.2)	910 (39.8)	0.503
	Yes	23 (46.9)	26 (53.1)		10 (52.6)	9 (47.4)	

Functional disability	No	3504 (64.9)	1894 (35.1)	0.008	1331 (60.3)	876 (39.7)	0.292
	Yes	89 (40.8)	129 (59.2)		31 (53.4)	27 (46.6)	

**Table 2:** Healthcare utilization by cross-sectional and incident pain.

Variable		Cross-sectional		*p* value	Incident		*p* value
		Overall pain			Overall pain		
		No	Yes		No	Yes	
Hospital in-patient	No	3247 (66.5)	1639 (33.5)	<0.001	1267 (62.8)	749 (37.2)	<0.001
	Yes	346 (47.4)	384 (52.6)		119 (41.2)	170 (58.8)	

Hospital out-patient	No	1976 (69.5)	868 (30.5)	<0.001	821 (65.6)	431 (34.4)	<0.001
	Yes	1617 (58.3)	1155 (41.7)		564 (53.7)	487 (46.3)	

Private clinic out-patient	No	3348 (65.1)	1792 (34.9)	<0.001	1218 (62.3)	737 (37.7)	<0.001
	Yes	245 (51.5)	231 (48.5)		167 (48.4)	178 (51.6)	

Traditional medicine practitioner	No	3538 (64.7)	1930 (35.3)	<0.001	1343 (61.2)	852 (38.8)	<0.001
	Yes	55 (37.2)	93 (62.8)		39 (37.5)	65 (62.5)	

		*Back pain*			*Back pain*		
Hospital in-patient	No	4365 (89.3)	521 (10.7)	<0.001	2495 (88.4)	327 (11.6)	<0.001
	Yes	589 (80.7)	141 (19.3)		352 (82.8)	73 (17.2)	

Hospital out-patient	No	2602 (91.5)	242 (8.5)	<0.001	1489 (88.9)	186 (11.1)	0.029
	Yes	2352 (84.8)	420 (15.2)		1357 (86.4)	214 (13.6)	

Private clinic out-patient	No	4550 (88.5)	590 (11.5)	0.018	2407 (88.8)	304 (11.2)	<0.001
	Yes	404 (84.9)	72 (15.1)		438 (82.3)	94 (17.7)	

Traditional medicine practitioner	No	4837 (88.5)	631 (11.5)	<0.001	2730 (88.3)	362 (11.7)	<0.001
	Yes	117 (79.1)	31 (20.9)		111 (74.5)	38 (25.5)	

		*Knee pain*			*Knee pain*		

Hospital in-patient	No	4057 (83.0)	829 (17.0)	<0.001	2124 (81.8)	473 (18.2)	<0.001
	Yes	517 (70.8)	213 (29.2)		262 (71.4)	105 (28.6)	

Hospital out-patient	No	2436 (85.7)	408 (14.3)	<0.001	1327 (83.7)	258 (16.3)	<0.001
	Yes	2138 (77.1)	634 (22.9)		1057 (76.7)	321 (23.3)	

Private clinic out-patient	No	4208 (81.9)	932 (18.1)	0.008	2011 (81.2)	465 (18.8)	0.035
	Yes	366 (76.9)	110 (23.1)		373 (77.1)	111 (22.9)	

Traditional medicine practitioner	No	4467 (81.7)	1001 (18.3)	0.004	2278 (80.9)	539 (19.1)	0.017
	Yes	107 (72.3)	41 (27.7)		101 (72.7)	38 (27.3)	

		*Leg pain*			*Leg pain*		

Hospital in-patient	No	4050 (82.9)	836 (17.1)	<0.001	2248 (86.8)	343 (13.2)	<0.001
	Yes	501 (68.6)	229 (31.4)		290 (78.8)	78 (21.2)	

Hospital out-patient	No	2433 (85.5)	411 (14.5)	<0.001	1385 (87.9)	191 (12.1)	<0.001
	Yes	2118 (76.4)	654 (23.6)		1151 (83.3)	230 (16.7)	

Private clinic out-patient	No	4183 (81.4)	957 (18.6)	0.030	2141 (86.4)	336 (13.6)	0.028
	Yes	368 (77.3)	108 (22.7)		394 (82.6)	83 (17.4)	

Traditional medicine practitioner	No	4442 (81.2)	1026 (18.8)	0.020	2423 (86.3)	384 (13.7)	<0.001
	Yes	109 (73.6)	39 (26.4)		107 (74.3)	37 (25.7)	

**Table 3:** Associations with cross-sectional pain, HART 2015.

Variables	CPR (95% CI)	*p* value	APR (95% CI)	*p* value
Age (in years)	1.014 (1.01 to 1.02)	<0.001	1.004 (0.998–1.011)	0.165
Female	1 (reference)	<0.001	1 (reference)	<0.001
Male	0.73 (0.65 to 0.81)		0.77 (0.67 to 0.88)	
≤Elementary education	1 (reference)	<0.001	1 (reference)	<0.001
>Elementary education	0.51 (0.44 to 0.59)		0.63 (0.53 to 0.76)	
Not married	1 (reference)	0.403	—	
Married	0.95 (0.85 to 1.07)			
Muslim or other	1 (reference)	0.182	—	
Buddhist	0.87 (0.71 to 1.07)			
Income high	1 (reference)	<0.001	1 (reference)	0.452
Income low	1.37 (1.23 to 1.53)		1.06 (0.92 to 1.21)	
Current alcohol use	0.84 (0.71 to 1.00)	0.049	1.06 (0.87 to 1.31)	0.558
Smoking tobacco	0.91 (0.77 to 1.08)	0.288	—	
Physical activity				
None	1 (reference)		—	
1–149 minutes/week	1.07 (0.94 to 1.22)	0.285		
≥150 minutes/week	0.94 (0.80 to 1.10)	0.438		
Body mass index				
Normal	1 (reference)		1 (reference)	
Underweight	1.21 (0.99 to 1.47)	0.057	1.10 (0.89 to 1.37)	0.380
Overweight	1.03 (0.87 to 1.21)	0.751	1.01 (0.84 to 1.20)	0.948
Obesity I	1.16 (1.00 to 1.35)	0.050	1.14 (0.97 to 1.35)	0.123
Obesity II	1.55 (1.23 to 1.95)	<0.001	1.28 (0.99 to 1.67)	0.061
Probable depression	1.74 (1.47 to 2.06)	<0.001	1.14 (0.93 to 1.41)	0.209
Self-reported mental health (poor)	1.63 (1.45 to 1.84)	<0.001	1.49 (1.29 to 1.72)	<0.001
Sleep problem	2.61 (2.26 to 3.01)	<0.001	1.99 (1.67 to 2.37)	<0.001
Arthritis or rheumatism	3.89 (2.93 to 5.17)	<0.001	3.29 (2.36 to 4.60)	<0.001
Hypertension	1.36 (1.21 to 1.52)	<0.001	1.13 (0.98 to 1.31)	0.103
Diabetes	1.20 (1.03 to 1.39)	0.019	0.90 (0.75 to 1.08)	0.278
Cardiovascular disease	1.54 (1.21 to 1.96)	<0.001	1.09 (0.81 to 1.47)	0.554
Kidney diseases	1.51 (1.02 to 2.22)	0.038	1.36 (0.86 to 2.13)	0.187
Brain diseases and psychiatric problems	2.88 (1.74 to 4.76)	<0.001	2.11 (1.12 to 3.96)	0.020
Lung diseases	2.02 (1.15 to 3.55)	0.014	1.94 (1.03 to 3.63)	0.039
Functional disability	1.46 (1.10 to 1.94)	0.008	1.03 (0.72 to 1.47)	0.879
Health care utilization				
Hospital in-patient	2.20 (1.88 to 2.57)	<0.001	1.88 (1.55 to 2.28)	<0.001
Hospital out-patient	1.35 (1.19 to 1.52)	<0.001	1.27 (1.10 to 1.47)	<0.001
Private clinic out-patient	1.76 (1.46 to 2.12)	<0.001	1.51 (1.20 to 1.90)	<0.001
Traditional medicine practitioner	3.10 (2.21 to 4.35)	<0.001	2.41 (1.60 to 3.63)	<0.001

CPR: crude prevalence ratio, APR: adjusted prevalence ratio, and CI: confidence interval.

**Table 4:** Associations with incident pain, HART 2015–2017.

Variables	CPR (95% CI)	*p* value	APR (95% CI)	*p* value
Age (in years)	1.02 (1.01 to 1.022)	<0.001	1.02 (1.01 to 1.03)	<0.001
Female	1 (reference)	<0.001	1 (reference)	0.035
Male	0.72 (0.61 to 0.85)		0.80 (0.66 to 0.99)	
≤Elementary education	1 (reference)	<0.001	1 (reference)	<0.001
>Elementary education	0.58 (0.46 to 0.72)		0.65 (0.51 to 0.83)	
Not married	1 (reference)	0.080	1 (reference)	0.553
Married	0.86 (0.72 to 1.02)		1.07 (0.86 to 1.33)	
Muslim or other	1 (reference)	0.008	1 (reference)	0.002
Buddhist	1.56 (1.12 to 2.17)		1.85 (1.26 to 2.73)	
Income high	1 (reference)	0.340	—	
Income low	1.09 (0.92 to 1.28)			
Current alcohol use	0.80 (0.63 to 1.03)	0.088	1.13 (0.83 to 1.47)	0.449
Smoking tobacco	0.62 (0.48 to 0.80)	<0.001	0.75 (0.55 to 1.03)	0.076
Physical activity				
None	1 (reference)		1 (reference)	
1–149 minutes/week	1.00 (0.82 to 1.22)	0.965	1.05 (0.85 to 1.31)	0.657
≥150 minutes/week	0.73 (0.58 to 0.93)	0.010	0.79 (0.62 to 1.02)	0.073
Body mass index				
Normal	1 (reference)		1 (reference)	
Underweight	0.91 (0.67 to 1.24)	0.566	0.84 (0.61 to 1.16)	0.278
Overweight	1.02 (0.81 to 1.30)	0.846	1.06 (0.83 to 1.37)	0.638
Obesity I	1.22 (0.97 to 1.53)	0.086	1.28 (1.01 to 1.62)	0.044
Obesity II	1.14 (0.78 to 1.68)	0.496	1.17 (0.78 to 1.75)	0.443
Probable depression	1.04 (0.78 to 1.40)	0.772	—	
Self-reported mental health (poor)	1.25 (1.04 to 1.52)	0.021	1.27 (1.02 to 1.58)	0.035
Sleep problem	1.27 (0.98 to 1.64)	0.075	1.11 (0.83 to 1.47)	0.486
Arthritis or rheumatism	1.19 (0.68 to 2.09)	0.547	—	
Hypertension	1.20 (1.01 to 1.43)	0.040	1.01 (0.82 to 1.25)	0.930
Diabetes	1.39 (1.10 to 1.75)	0.006	1.13 (0.86 to 1.48)	0.397
Cardiovascular disease	1.30 (0.88 to 1.94)	0.191	—	
Kidney diseases	1.79 (0.93 to 3.44)	0.080	1.63 (0.83 to 3.22)	0.159
Brain diseases and psychiatric problems	1.32 (0.48 to 0.366)	0.591	—	
Lung diseases	1.36 (0.55 to 3.36)	0.504	—	
Functional disability	1.32 (0.78 to 2.23)	0.294	—	
Healthcare utilization				
Hospital in-patient	2.42 (1.88 to 3.11)	<0.001	1.75 (1.31 to 2.35)	<0.001
Hospital out-patient	1.65 (1.39 to 1.95)	<0.001	1.21 (0.98 to 1.50)	0.074
Private clinic out-patient	1.76 (1.40 to 2.22)	<0.001	1.59 (1.22 to 2.07)	<0.001
Traditional medicine practitioner	2.63 (1.75 to 3.34)	<0.001	2.57 (1.61 to 4.09)	<0.001

CPR: crude prevalence ratio, APR: adjusted prevalence ratio, and CI: confidence interval.

## Data Availability

The data source is publicly available at Gateway to Global Ageing Data, Health, Aging, and Retirement in Thailand: https://g2aging.org/?section=study&studyid=44.
